# Structure–Function Correlations in Sputter Deposited Gold/Fluorocarbon Multilayers for Tuning Optical Response

**DOI:** 10.3390/nano9091249

**Published:** 2019-09-03

**Authors:** Pallavi Pandit, Matthias Schwartzkopf, André Rothkirch, Stephan V. Roth, Sigrid Bernstorff, Ajay Gupta

**Affiliations:** 1 Deutsches Elektronen-Synchrotron (DESY), Notkestraße 85, D-22607 Hamburg, Germany; 2 KTH Royal Institute of Technology, Department of Fibre and Polymer Technology, Teknikringen 56-58, SE-100 44 Stockholm, Sweden; 3 Elettra-Sincrotrone Trieste, SS 14, Km 163.5, I-34149 Basovizza, Trieste, Italy; 4 Center for Spintronic Materials, Amity University, UP Noida 201 313, India

**Keywords:** nanocomposite, metal–polymer interface, multilayer, structure–function correlation, indirect band gap, GISAXS, GIWAXS, UV-Vis

## Abstract

A new strategy to nanoengineer gold/fluorocarbon multilayer (ML) nanostructures is reported. We have investigated the morphological changes occurring at the metal–polymer interface in ML structures with varying volume fraction of gold (Au) and the kinetic growth aspect of the microscale properties of nano-sized Au in plasma polymer fluorocarbon (PPFC). Investigations were carried out at various temperatures and annealing times by means of grazing incidence small-angle and wide-angle X-ray scattering (GISAXS and GIWAXS). We have fabricated a series of MLs with varying volume fraction (0.12, 0.27, 0.38) of Au and bilayer periodicity in ML structure. They show an interesting granular structure consisting of nearly spherical nanoparticles within the polymer layer. The nanoparticle (NP) morphology changes due to the collective effects of NPs diffusion within ensembles in the in-plane vicinity and interlayer with increasing temperature. The in-plane NPs size distinctly increases with increasing temperature. The NPs become more spherical, thus reducing the surface energy. Linear growth of NPs with temperature and time shows diffusion-controlled growth of NPs in the ML structure. The structural stability of the multilayer is controlled by the volume ratio of the metal in polymer. At room temperature, UV-Vis shows a blue shift of the plasmon peak from 560 nm in ML Au/PTFE_1 to 437 nm in Au/PTFE_3. We have identified the fabrication and postdeposition annealing conditions to limit the local surface plasmon resonance (LSPR) shift from $\Delta \lambda_{LSPR}$ = 180 nm (Au/PTFE_1) to $\Delta \lambda_{LSPR}$ = 67 nm (Au/PTFE_3 ML)) and their optical response over a wide visible wavelength range. A variation in the dielectric constant of the polymer in presence of varying Au inclusion is found to be a possible factor affecting the LSPR frequency. Our findings may provide insights in nanoengineering of ML structure that can be useful to systematically control the growth of NPs in polymer matrix.

## 1. Introduction

Easy processability, high flexibility, and tunable physical properties make nanocomposites very attractive for a broad range of applications. Recently, the combination of metal nanoparticles with dielectric media, such as polymers, has gained great pertinence both in fundamental as well as technological aspects. Their fine control and possible tuning of physical properties can lead to the fabrication of materials with novel functional, electric, and optical properties and engender their accessibility to various applications in the field of optics, electronics, and biomedicine [1,2,3,4,5]. These physical properties of the metal are strongly morphology-dependent [6,7,8,9]. In particular, the metal undergoes a significant property change compared to the bulk due to the quantum confinement towards nanoscale and their large surface to volume ratio. Interfacial energy variation may also enhance their functionality [1]. Incorporation of metallic nanoparticles (NP) into a polymer improves the functionality of the polymer. Governed by the metal–polymer interactions, which generally differ from the polymer–polymer interactions, the properties of the composite material are thus dominated by their interfacial interactions [10,11,12,13]. The morphologies of the embedded metal NPs can be artificially modified by controlling their processing parameters, such as the preparation method, rate of deposition, thermal annealing, etc. [6,9,10,14]. Furthermore, the volume fraction of the metal in the polymer matrix plays a significant role in deciding the morphological structure. The conductivity of metal–polymer nanocomposites (MPNC) varies from insulating to conducting as a function of metal concentration. The resistivity drops by several orders of magnitude near percolation [15]. The conductivity depends exponentially on the cluster separation near the percolation threshold (insulator metal transition), which is proved by varying the metal–polymer volume fraction $\varphi =\frac{V_{m}}{V_{p}}$, with $V_{m}$ & $V_{P}$ being the volume of metal and polymer, respectively. Furthermore, in-plane growth of NPs and their effect on the properties is limited by the percolation threshold as the metal layer above the percolation threshold gains a three-dimensional structure [1,6,16]. Varying the metallic volume fraction in the dielectric matrix also influences the refractive index and this can alter the optical properties of nanocomposite [17]. Additionally, thermal annealing of MPNC can artificially modify the morphological structure due to enhancement of atomic mobility and diffusivity at higher temperature [18]. The resulting structure of NPs is then driven by nucleation, thermal mobility, and growth. However, the fabrication and the control of the growth of NPs for desired applications of such materials is still a major challenge. To study the individual particle growth, emphasis has been given to the MPNC multilayer (ML) structure of fixed volume fraction below the percolation threshold of metal in varying polymer layers. Such arrangement allows inter alia control growth of NPs in the polymer matrix. MPNC MLs have been prepared via sputter deposition, having nanofabrication capability of sequential deposition of metal and polymer with the same precision [16,19]. Particularly, gold nanoparticles (Au NPs) modified with plasma polymer fluorocarbon (PPFC) [20] (sputtered Polytetrafluorethylene (PTFE)) MLs have been prepared by alternating sputtering from an Au and a PTFE target. Au NPs in the PPFC matrix show long-term stability, brilliant optical properties, and lead to a spectral optical shift upon variation in their size, shape, and surrounding dielectric matrix [21]. PTFE has high chemical stability and comparatively high glass transition temperature with heat resistance capacity. It has low surface free energy that supports growing spherical NPs and a high sputter yield with low crosslinking tendency [22], making this polymer especially suitable for sputter deposition and for such studies. The correlation of the Au NPs’ morphology with the resulting optical properties of the MLs has been investigated in this study. The structural properties of the nanocomposites and kinetic growth of NPs in polymer matrix have been studied using grazing incidence small and wide-angle X-ray scattering (GISAXS & GIWAXS). GISAXS yields the statistically pertinent horizontal and vertical correlation information of the changing electron density distribution [23,24] during growth, while GIWAXS can provide information about NPs’ crystallinity and kinetic growth of NPs in polymer [25,26]. UV-Vis spectroscopy has been used to study the optical properties of nanocomposites [27,28]. The optical behavior of the nanocomposite evolves upon metal volume fraction variation due to the rearrangement of the NPs in the polymeric matrix. The range of the plasmon peak shift depends upon the density of implanted NPs and the thickness of the intermediate polymer layer. To further contextualize the structure–function correlations, we have performed isochronal thermal annealing of the ML structure and then compared the growth tendencies to the optical behavior. Hereby, the first question addressed is the effect of the metal volume fraction on the structural and optical properties of the nanocomposite. The second is the effect of temperature-induced Au NP growth behavior in PPFC matrix. The relation of structural and optical properties during this growth is discussed.

## 2. Experimental Details

### 2.1. Sample Preparation

The ML structure was prepared on optically polished Si (100) substrate (Si-Mat, Kaufering, Germany). Prior to the deposition, the Si wafers were ultrasonically cleaned with acetone, isopropanol, and deionized water for 10 min, each followed by piranha cleaning. This strongly oxidizing chemical cleaning removes most organic matter and ion contamination from the substrate [29]. MPNC MLs were prepared by radio frequency (rf) ion beam sputtering, using two alternating independent gold (Mateck, Berlin, Germany, purity 99.99%) and PTFE targets (Disk 2800 g/mol; Science Fellow Industries, Indore, India, purity 98%). Ion bombardment was initiated by a plasma glow discharge using a 3 cm broad-beam Kaufman-type hot-cathode at a low argon pressure (2.5 sccm), which thus generates Ar ions. The base pressure in the chamber was 3 × 10^−7^ mbar. The chamber was flushed with pure Ar (99.995%) a few times before deposition to minimize oxygen and water vapor contamination. The accelerating voltage and current were 1000 V and 30 mA, respectively. The target was kept at 45° with respect to the argon beam direction. The substrate was kept parallel to the target at a distance of 150 mm. The deposition pressure was 2.6 × 10^−3^ mbar. A schematic diagram for sputtering setup involved is shown in Figure S1. Three nanocomposites were prepared by varying the Au volume fraction. The deposition rate of each of the materials (Au and PTFE) was determined separately at the same pressure and gas flow rate which was used for alternate sputtering. Deposited PTFE is named hereafter plasma polymer fluorocarbon (PPFC), due to its structural and chemical changes during sputtering. The effective thickness rates monitored were *J_Au_* = 0.92 ± 0.03 Å/s and *J_PTFE_* = 3.4 ± 0.2 Å/s. The filling factor of gold in three samples was 0.12, 0.27, and 0.38, which is below the gold percolation threshold of 0.45 as reported for gold in amorphous fluoroplastics [15]. For the fixed filling factor, nanocomposite density and volume fraction were calculated in terms of bilayer period in three MLs. The volume fraction was estimated by considering the film thickness ratio of Au and PTFE inside the ML structure. The thickness estimation for the various volume fractions has been detailed in the SI. The in-plane thickness of each gold layer was kept constant at 1.0 ± 0.1 nm in the entire three MLs, whereas the thickness of the polymer layer varied in the three samples (as 19.0, 9.0, and 5.6 nm, respectively) to offer different volume fractions of Au in PPFC. The total thickness of the ML stack in all three cases was 140 ± 5 nm. The structures of the films were as follows: Si/SiO_2_/[Au(1 nm)/PPFC(19 nm)]_7_; [Au(1 nm)/PPFC(9 nm)]_14_ and [Au(1 nm)/PPFC(5.6 nm)]_21_. The indices in subscript denote the total number of bilayers in the three MLs. The samples named thereafter are Au/PPFC_1, Au/PPFC_2, and Au/PPFC_3. The schematic arrangement of NPs in the three MLs is shown in (SI) Figure S2a–c, respectively.

### 2.2. Characterization

A monochromatic X-ray beam with a wavelength of 0.154 nm was used for the simultaneous GISAXS and GIWAXS measurements at the SAXS beamline (BL 5.2), Elettra synchrotron, Trieste [30]. The incident beam had a cross section of 1.2 mm (H) × 100 μm (V). The small angle scattering signal was recorded with a 2D image plate detector (MAR 300; pixel size of 150 × 150 μm^2^). The image plate was kept in the forward direction at a distance of 1850 ± 1 mm from the sample, to measure the scattered intensity in forward direction. The intensity of the incident and specular beam near *q_y_* = 0 nm^−1^ was attenuated with a partly transparent aluminum filter in the beam path. Measurements were performed at constant grazing incidence angle α_i_ ~ 0.45°. For in-plane GIWAXS measurements, a Pilatus 100 K (Dectris Ltd., Baden, Switzerland; pixel size of 172 × 172 μm^2^) detector was kept at a distance of 327.7 ± 0.5 mm in the film plane (tilted 55°; a sketch of the detector position used for experiment is shown in Figure S3.) This detector distance had been fixed to cover the required angular range. The exposure time for each measurement was set to 300 s for GISAXS and GIWAXS. To address the possible effects of X-ray irradiation on the structure, the ML was initially exposed to the photon beam for a longer time: up to 20 min exposure, no significant change in the scattering pattern was observed. Beyond 20 min, a small broadening of the diffraction peaks and a decrease in peak intensities were observed. Accordingly, to avoid any effect of radiation damage during the measurements, after every scan the sample was shifted across the beam by 2 mm (sample size 20 × 20 mm^2^) to expose an unexposed area (fresh area) of the film. The angular detector ranges have been converted from pixel to the scattering vector q using as two standard samples calibration reference; rat tail tendon collagen for GISAXS and Cu foil for GIWAXS. The width of the calibrant Cu diffraction lines (*W_ins_*) was used as instrumental width for estimating the experimental crystalline size in the GIWAXS data of MLs using the Debye–Scherrer formula [31]. The MLs were isochronally annealed at 373 K, 473 K, and 573 K in situ during the measurement to study the kinetic growth of particles with time and temperature. A miniature boron nitride (BN) furnace (mounted on the sample rotation stage) was used for controlled heating of the sample, which was kept in a protective atmosphere of nitrogen gas. The sample temperature was maintained with an accuracy of ±0.5 K. The optical reflectance spectra of the MPNCs were measured ex situ with a UV-Vis spectrophotometer (Perkin Elmer, Waltham, WA, USA. Model: Lambda 950). The scans (normal incidence; reflectance mode) were recorded in the wavelength range of 200 nm to 800 nm. The main interest was to see the variation in the surface plasmon resonance of Au.

## 3. Results and Discussion

### 3.1. Structural Properties at Room Temperature

**Figure 1:**
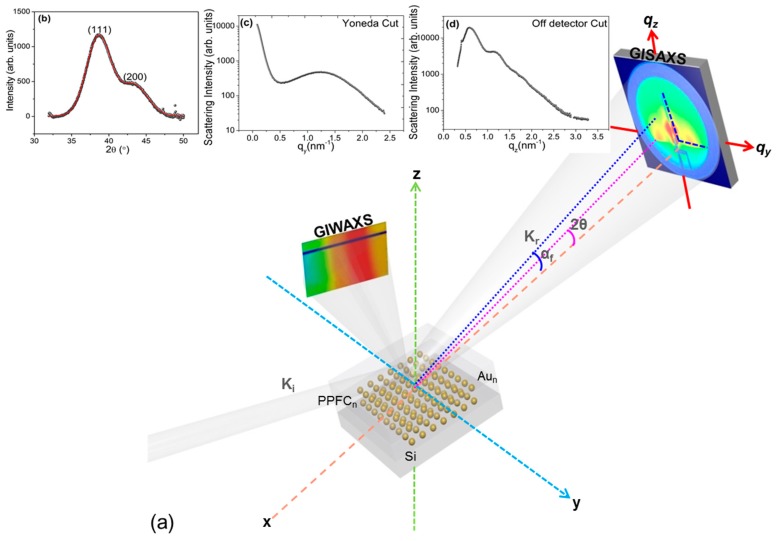
(**a**) Schematic view of the GISAXS experiment combined with in-plane-GIWAXS; (**b**) the extracted 1D GIWAXS profile was taken as line cut as indicated in the 2D GIWAXS image, (**c**) Yoneda cut (*q_y_* = 0.236) and (**d**) off-detector cut (along *q_z_*) as indicated by blue dashed lines in the 2D GISAXS pattern. In the GISAXS pattern, the origin of the coordinates of *q_y_* and *q_z_* is indicated by the direct beam position.

An overview of the setup used for simultaneous GISAXS and GIWAXS measurements at the SAXS beamline (BL 5.2) Elettra, Trieste and their extracted 1D information is shown in Figure 1.

### 3.2. GIWAXS

The 1D diffraction pattern (Figure 1b) was extracted from the 2D in-plane diffraction image of the sample in θ−2θ geometry. The GIWAXS pattern confirms the formation of well-established nanocrystalline particles due to the appearance of two diffraction peaks around 2θ = 38.60 ± 0.008° and 43.30 ± 0.003°, ascribed to the (111) and (200) planes of fcc crystalline Au [32]. The peak positions are shifted to some extent from the bulk position which reflects the strain that may be caused by the geometry and the polymer–filler interfacial interaction. Two Gaussian peaks (red line in Figure 1b) were fitted to the data and the minimum crystalline size of a NP ($D_{np}$) was derived from the width obtained for the (111) peak in the diffraction pattern, after deducting the instrumental width using the Debye–Scherrer formula [31]:

(1) $$D_{np}=\frac{0.94\lambda }{B\;\cos\theta },\;B=\sqrt{W_{E}^{2}-W_{ins}^{2}}$$

where *λ* is the wavelength of the X-rays and *B* is the full width at half maximum in radians. W_E_ and *W_ins_* (0.35 rad.) are the experimental and instrumental width, respectively. The lattice constant nearly matches with the bulk value of Au (0.408 nm) which confirms crystalline gold NPs in the polymer matrix. Results obtained are given in Table 1. Diffraction plots of all three samples at room temperature are shown in the Figure S2d. As one can see from Table 1, the crystalline size in all three MLs is almost the same despite of the varying volume fraction of gold in ML. This is as per expectation, since the deposited in-plane mass thickness of the individual gold layers is the same (1.0 ± 0.1 nm) for all samples and the relative metal content is varied only by varying the thickness of the polymer layers.

### 3.3. GISAXS

**Figure 2:**
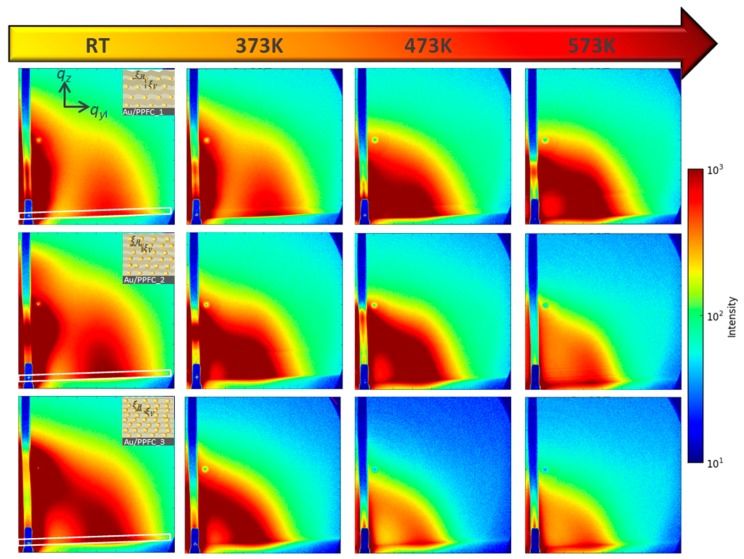
GISAXS pattern of MLs (**a**) Au/PPFC_1, (**b**) Au/PPFC_2, and (**c**) Au/PPFC_3 at the indicated temperatures. The white rectangle block in all three ML’s room temperature patterns shows the projected area chosen to extract structural lateral features after applying 2° tilt correction. The inset of GISAXS room temperature images of three MLs shows the schematic layer arrangement and their horizontal $\left(\xi_{H}\right)$ and vertical order ($\xi_{V})$.

In-plane cuts I(*q_y_*) were made at the Yoneda peak of Si (*q_z_* = 0.236 nm^−1^) and the DPDAK software package [33] was used to extract quantitative information from the data sequence. The last image taken at a particular temperature is illustrated in Figure 2. Please note that a small tilt can be observed in the measured 2D pattern which we associate to an initial tilt in the heater assembly on which the specimen was mounted (see Figure 1 for details of the setup). Tilt was accounted for when making the cuts, see indicated area (white block) in Figure 2, first left column.

The GISAXS pattern shows a broad side peak that emerges at large *q_y_* values (Figure 1c). This peak is related to a maximum interference of scattered waves describing the NP correlation distance, often called interparticle distance $\xi_{H}$ [16,25,34]. From the position of the side peak (horizontal) one can estimate the average interparticle distance ($\xi_{H}$) using the following formula [16],

(2) $$\xi_{H}\approx \frac{2\pi }{q_{y}}$$

where *q_y_* is the position of the side peak in the *y* direction. In the Yoneda cut I(*q_y_*) at constant *q_z_* = 0.236 nm^−1^, the presence of a peak (Figure 1c) evidences that the island distribution is not completely random, but are separated by a preferential nearest neighbor distance ξ (center-to-center), indicating short range ordering of Au NPs in PPFC. The side maxima observed for pristine MLs are dominated by the structure factor signals and indicate a substantial lateral ordering of the NPs. The peak in the *q_y_* range of 1.45–1.46 nm^−1^ corresponds to the interparticle distance $\xi_{H}$ ≈ 4.3 ± 0.05 nm in real space, obtained by simulation (see SI for details). Simulation of Si/[Au(1 nm)/PPFC(19 nm)] was done using distorted wave born approximation (DWBA). The error in interparticle distance ($\xi_{H}$ ≈ 4.3 ± 0.05 nm) is the fitting error of average size and distance. Indeed, the GISAXS pattern shows a broad distance variation of 25% which was also used in the simulation. Yoneda cuts of three pristine samples are shown in Figure S2e. The same interparticle distances $\xi_{H}$ were obtained for all three samples (see Table 1), which corroborates a constant in-plane thickness of gold resulting in a uniform interlayer morphology. Similar to Equation (2), the vertical interlayer-particle distance $\xi_{V}$ was derived using,

(3) $$\xi_{V}\approx \frac{2\pi }{q_{z}}$$

where *q_z_* denotes the position of the first order Bragg peak in the off-detector cut.

The off-detector cut demonstrates the vertical ordering in MPNC, executed (along *q_z_*) for 1.85 nm^−1^ < *q_y_* < 1.89 nm^−1^ (Figure S3d). In the vertical direction, an estimable interlayer interparticle correlation is observed due to the periodic structure of the MPNC ML (Table 2). As the first Au layer is deposited on the Si wafer, Au NPs are formed which exhibit in-plane local ordering of a granular nanoclusters assembly [16,35]. The next deposited PPFC amorphous layer perfectly covers the Au NPs layer. The successive alternate deposition of Au and PPFC layers leads to the appearance of small undulations/waviness with a period order given by the vertically interlayer-particle distance ($\xi_{V})$, see also Figure S2a. Layer-by-layer self-organized growth of NPs is the origin of this vertical order. The vertical order leads to well defined Bragg peaks in the scattering intensity from the MLs. The Bragg peaks in the vertical direction show the strong structural coherence between the individual layers of the MLs [36,37,38]. The off-detector cuts of three pristine MLs are given in Figure S2f. Vertically, the Bragg peak is shifted towards higher q values from sample Au/PPFC_1 to Au/PPFC_3. The bilayer period of Au/PPFC_1 to Au/PPFC_3 was varied in order to obtain different volume fractions of gold in the total ML stack of three MLs (Figure S2a–c). Thus, the shift in the Bragg peak position signals the fact that as the PPFC layer thickness decreases from Au/PPFC_1 to Au/PPFC_3, the separation of the Bragg peaks increases (due to reduce periodicity in successive MLs.; Figure S2f). The presence of a successive number of Bragg peaks in the GISAXS pattern of the MLs indicates that the NPs in the layers are well separated and the metal–polymer period is rather well established. In addition, the peaks broaden from sample Au/PPFC_1 to Au/PPFC_3 (Figure S2f). This can be explained as the interfaces are not sharp, but rather show a diffuse compositional profile which is evidence of a strong intermixing of metal and polymer at the interfaces in the pristine sample. This can be understood in terms of interface modulation [39] and stress in geometry of the three MLs according to the volume fraction of Au in PPFC. In this direction, Amarandei et al. reported that in MPNCs, the Au NPs interactions with the underlying layers must be considered [34,40]. For MLs Au/PPFC_2 and Au_PPFC3, the polymer thickness is comparable (low enough) to NPs size, which thermodynamically favors the interlayer interaction of NPs [41]. The undulated structure of the PPFC films increases with decreasing PPFC layer thickness because the thinner PPFC layer covers the waviness of the Au layers less efficiently (see schematic structures shown in Figure S2a–c). Consequently, the embedding of the Au inclusions in the intervening PPFC matrix increases and the vertical coherence length decreases [38]. We further note that the intensity of the first Bragg peak (indicated by arrow in Figure S2f) depends on the effective interdiffusivity/embedding in the MLs.

The interlayer-particle distance ($\xi_{V})$ decreased from ML Au/PPFC_1 to Au/PPFC_3 (from 10.5 to 3.8 nm) due to the decrease in the period in successive MLs. This is in conformity with the constant in-plane gold thickness and varying polymer thickness in the three successive MLs. From the off-detector cuts of pristine MLs (as given in Figure S2f), the structure and morphology of the NPs in a vertical stack can be estimated. For extracting the lateral distance $\xi_{H}$, we performed a simulation of the GISAXS signal in the Yoneda cut using the software IsGISAXS [42]. Details are described in Figure S4.

### 3.4. Structural Changes during and after Annealing

**Figure 3:**
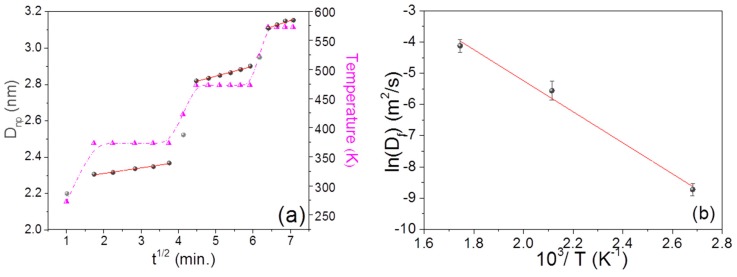
(**a**) Crystalline size (from GIWAXS) variation in Au/PPFC_3 as a function of annealing time $\left(\sqrt{t}\right)$ at varying temperature; the dotted (pink) curve represents the temperature profile of the sample with time and temperature (**b**) Arrhenius plot showing the relationship between ln(*D_f_*) (where, *D_f_* is the diffusion constant) and 10^3^/T for the Au/PPFC_3 ML obtained from in situ temperature-dependent in-plane GIWAXS.

***GIWAXS:*** Annealing of the nanocomposite thin films well above the glass transition temperature (T_g_) reduces the interaction bond strength between metal NPs and polymer molecules. The ML structure undergoes reorganization because of the change in the physical structure of the polymer at higher temperatures. The mobility of the NPs increases due to weaker interface interaction, accompanied by a weaker entanglement density of the polymer chains. As a result, NPs start to diffuse through the polymer network. More and more NPs come in contact with each other, and eventually coagulation of these particles occurs [18,43]. This would increase the average crystalline size/NP size, interparticle distance, and may modify NPs morphology. As mentioned earlier, to study the effect of temperature on the nanocomposite properties, isochronal thermal annealing of the MLs was performed at various temperatures. A number of scattering patterns were recorded in a period of 20 to 30 min. with exposure time of 298 s at intervals of 5 min for statistical analysis. Thus, at each temperature, a number of scattering patterns were recorded as a function of time. To estimate the kinetic behavior of the NPs in the polymer matrix, the diffusion of NPs was estimated using the equation of diffusion [44,45],

(4) $$D_{np}{}^{2}=2D_{f}t$$

where $D_{np}$ denotes the minimum crystalline size (Equation (1)), $D_{f}$ is the diffusion constant and *t* is the annealing time. Figure 3a shows the variation of minimum crystalline size in Au/PPFC_3 as a function of $\sqrt{t}$ at different annealing temperatures. One can see that the crystalline size increases linearly as a function of $\sqrt{t}$ at all investigated temperatures, suggesting diffusion-controlled growth of NPs in the polymer matrix at the varying temperatures. The slope of the straight line is proportional to the diffusivity of NPs in the polymer matrix. The variation in crystalline size was found to be systematic in all the samples (see Figure S5a,b). The obtained values of ‘$D_{f}$’ are used to find the activation energy ($E_{a}$) from Equation (5) [38,46]

(5) $$D_{f}=D_{0}\exp\left(\frac{-E_{a}}{RT}\right)$$

where R is the gas constant, $D_{0}$ is a pre-exponential factor, and T is the annealing temperature. From the slope of ln ($D_{f})$ vs. 10^3^/T plot (shown in Figure 3b), the activation energy of Au NPs interdiffusion in Au/PPFC_3 is calculated as $E_{a}=$ (0.34 ± 0.02 eV). Arrhenius plots of ML Au/PPFC_1 and Au/PPFC_2 are given in Figure S5c,d.

The glass transition temperature of bulk PTFE is around 394 K. It is interesting to see from Figure 3b that even at 373 K, which is below the glass transition temperature, the diffusion of NPs in the polymer matrix is governed by the same activation energy as evidenced by the Arrhenius plot. A change in viscosity across the glass transition temperature is expected to result in a significant change in the diffusion mechanism of NPs. The NP diffusion at all the three temperatures studied in the present work is governed by the same activation energy and suggests that no glass transition takes place in the temperature range investigated. The glass transition temperature is known to get reduced in the thin film form or with the inclusion of nanofillers [47,48]. Thus, it is quite likely that in the present system, the glass transition temperature has gone below 373 K (well above RT (273 K) but less than 394 K). The activation energy decreased from Au/PPFC_1 (1.03 ± 0.02) eV to Au/PPFC_3 (0.340 ± 0.02) eV [Au/PPFC_2 (0.429 ± 0.02)]. This is due to the fact that the polymer intermediate barrier layer between the two Au layers was successively decreased in the three MLs. Thus, the polymer layer mass density is in decreasing order, causing a decrease in activation energy.

**Figure 4:**
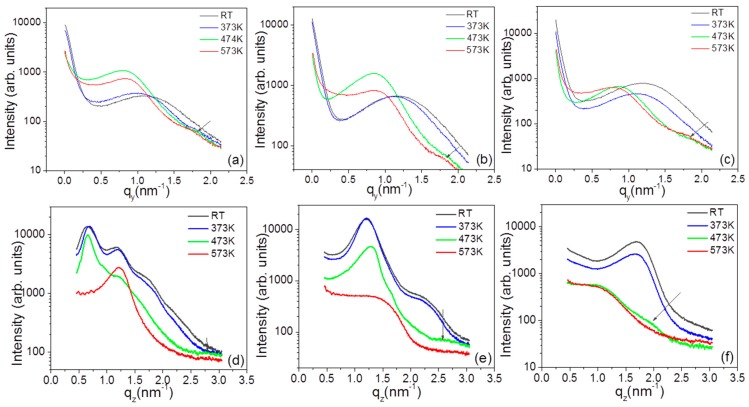
Directional cuts obtained from MLs at various temperatures. Yoneda cuts (horizontal) are shown in (**a**–**c**) and off-detector cuts (vertical) are shown in (**d**–**f**) for the MLs Au/PPFC_1, Au/PPFC_2, and Au/PPFC_3, respectively. In each plot, the arrow at higher q values denotes the presence of a higher order peak.

***GISAXS:*** The film morphology at various temperatures was accessed with GISAXS. The 2D GISAXS patterns recorded at various temperatures are shown in Figure 2. The Yoneda peak is accompanied by a broad side peak at higher *q_y_*. Figure 4a–c gives the Yoneda cuts made for the three MLs at different temperatures The lateral correlation peak in Au/PPFC_1 and Au/PPFC_2 is continuously shifting towards lower *q_y_* up to 473 K and then slightly shifts to higher *q_y_* at 573 K signalizing first an increase in the interparticle distance of the NPs and then a slight decrease at 573 K. In Au/PPFC_3, the interparticle distance is continuously increasing with temperature up to 573 K. In the ML structure, the diffusion of NPs at lower temperature is mostly confined within the Au layers, i.e., parallel to the substrate. However, the NPs gain higher mobility in both horizontal and vertical directions with increasing temperature. At 573 K, the polymer is close to its molten state and the NPs thus gain more freedom in both directions. The maximum scattering intensity related to the NPs layer rapidly moves towards lower *q_y_* at 573 K, indicating a change in the interparticle distance $\xi_{H}$. Thus the interparticle distance at higher temperature is due to the NPs mobility in both directions. However, Au/PPFC_3 has the lowest polymer layer thickness; due to this insufficient intermediate polymer thickness, it does not completely separating the Au NP layers. Hence, the NPs achieve the highest mobility at a lower temperature and the interparticle distance continuously increases as a collective response of the mobility of NPs in both vertical and horizontal directions in the ML. It is worth mentioning that at higher temperature, a higher order peak at larger *q_y_* values (marked in Figure 4) occurs. This indicates a more ordered state of the structure due to the thermal annealing.

The in-plane average NPs radius R was derived from the effective layer thickness (*δ*) of gold and the interparticle distance based on the geometrical model by Schwartzkopf et al. [16] by applying it to spherical NPs. The geometrical model assumes that the effective deposited material is locally separated into a hexagonal array of spherical clusters in a distance $\xi_{H}$. According to this model assumption, the average radius of the supported clusters can be calculated by:

(6) $$R=\sqrt[{3}]{\frac{3^{1.5}}{8\pi }\xi_{H}{}^{2}\delta }$$

With effective thickness δ=1.0±0.1 nm of Au and interparticle distance $\xi_{H}$. The resulting sizes of the NPs for different temperatures are given in Table 3.

**Figure 5:**
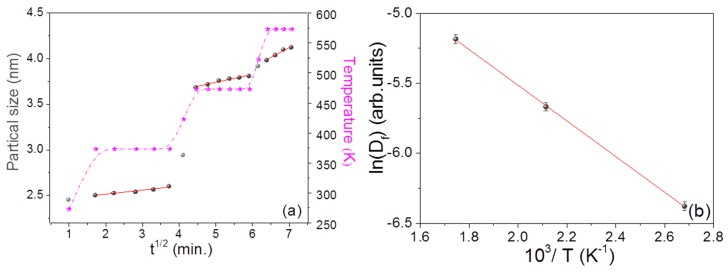
(**a**) Particle size variation in Au/PPFC_3 as a function of annealing time $\left(\sqrt{t}\right)$ at varying temperature; the dotted (pink) curve represents the temperature profile of the sample with time and temperature (**b**) Arrhenius plot showing the relationship between ln(*D_f_*) and 10^3^/T for the Au/PPFC_3 ML obtained from in situ temperature-dependent in-plane GISAXS; red line showing linear fit.

Particle radius slightly decreased in MLs Au/PFC_1 and Au_PPFC_2 from temperature 473 K to 573 K. This might be due to slight form change. Particle growth at various temperatures and their corresponding activation energy (0.109 ± 0.01 eV) for interdiffusion of adjacent NPs through the polymer matrix is shown in Figure 5a,b. One can observe a decrease in activation energy from Au/PPFC_1 (0.572 ± 0.01 eV) to Au/PPFC_2 (0.129 ± 0.01 eV) in Figure S6a,b. The activation energy is found to be lower in the analysis of particle growth by GISAXS than when using minimum crystalline size estimation via GIWAXS. The linear incremental trend in the activation energy plot is same in both calculated from crystalline size (GIWAXS) and particle size (GISAXS). The results are consistent, as the minimum crystalline size is lower than the particle size and it increases with temperature.

Off-detector cuts were also made from the data to more carefully analyze the changes occurring during annealing; they are shown in Figure 4d–f. One can notice that at 373 K there is no significant change in the Bragg peak position in all three MLs, showing that the ordering is preserved below the glass transition temperature (T_g_) (one should expect change in morphology crossing glass transition temperature), although there is small decrease in intensity due to the gradual mixing at the interfaces. However, a further increase in temperature to 473 K results in a significant change in the structure. At 473 K, the first Bragg peak in Au/PPFC_1 ML is slightly shifted towards lower *q_z_*, while for other two MLs the Bragg peak is shifted towards higher *q_z_* and the higher order Bragg peaks become suppressed. This might be because of a rearrangement of the NPs in PPFC leading to a more compact structure of the ML with higher intermixing at the metal–polymer interface. A further increase in temperature results in a rapid decrease in interlayer particle distance ($\xi_{V})$. Although $\xi_{V}$ is decreased remarkably at 573 K, the presence of a broad Bragg peak or hump shows that the layered structure is sustained. The Bragg peak is increasingly broadening from ML Au/PPFC_1 to Au/PPFC_3 as a result of higher intermixing at elevated temperature. This transformation shows that the MLs are converted into a diffused metal–polymer structure at 573 K. The interlayer particle distance of the MLs at various temperatures is listed in Table 2.

### 3.5. Optical Properties at Room Temperature

**Figure 6:**
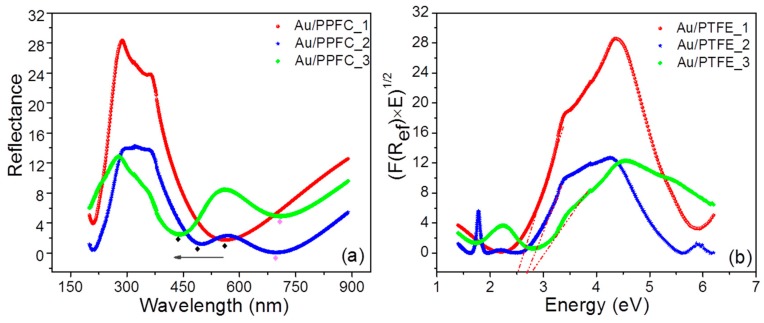
(**a**) UV-Vis spectra of three Au/PPFC MPNCs at room temperature; the local surface plasmon resonance (LSPR) in the three MLs are indicated by a diamond symbol (♦) and the arrow indicates the shift in LSPR. (**b**) Plot of [$F\;\left(R_{ef}\right)$ × (hν)] vs. energy for indirect band gap calculation by applying linear approximations (fits are indicated by the red dash-dotted lines).

Ordered NPs are promising structures that enable interconversion of the propagation of electromagnetic waves and thereby promote strongly enhanced local fields used for a number of practical applications such as photonics, optical sensors, etc. [43,49,50]. UV-Vis spectra of the three pristine MLs are shown in Figure 6. The spectra have been taken in off-specular reflection mode; dips in the spectra are due to absorption. One can notice characteristic features associated with NP assembly and their arrangement in the ML structure. In the wavelength range of 250 nm to 400 nm, some interference is seen [51] because of the ML structure. In the higher wavelength region, a surface plasmon is emerging in the spectrum, indicated by the diamond symbols in Figure 6. UV-Vis spectra of the nanocomposites show strong absorption in the range of 440 nm to around 520 nm. In the present case, we will refer to this as local surface plasmon resonance (LSPR) because in ML structures, NPs layers are buried in the polymer matrix. One can notice the presence of a single plasmon resonance in ML Au/PPFC_1 while for Au/PPFC_2 and Au/PPFC_3, the plasmon resonance splits in two.

A single broad Gaussian plasmon dip in the reflection spectra can be an indication of spherical NPs [52,53]. The analysis has been done considering the spherical shape of the NPs, which was also confirmed by the GISAXS data. The polarizability is a function of the inclusion geometry (independent of volume) and orientation with respect to the applied electrical field, termed as the depolarization factor. The optical study shows that the electric field distribution across the surface of the spherical particle seems to be uniform, and thus all the free conduction electrons oscillate in phase, resulting in one plasmon resonance, regardless of the type of incident polarization [54,55]. The isotropic optical response of the spherical particles can be altered either by increasing their aspect ratio (changing the particles morphology) or coupling, i.e., placing them in close proximity of other particles [50,56,57,58]. In Au/PPFC_1, the periodic polymer thickness is 19 nm which ensures that the voids between NPs become completely filled with PPFC. The structurally continuous polymer barrier layer completely separates the metallic layer vertically, which in turn hinders the effective movement of NPs in the vertical direction (Figure S2a). Thus, an ultrafine constant thickness of 1 nm of gold ensures local monodispersion of in-plane particles with small size variation which supports the appearance of a single plasmon in Au/PPFC_1. Local monodisperse distribution of NPs has also been confirmed by GISAXS. In the other two MLs, because of the lower thicknesses of polymer (9 nm and 5.6 nm, respectively), the polymer film is not continuous, but PPFC grows preferentially within the voids between neighboring particles. The over layer PPFC thickness is not capable of complete isolation of metal NPs in a vertical stack. As a consequence, the NPs distance becomes small enough to give rise to plasmon coupling between the interacting particles due to close proximity, which changes the distribution of the induced surface dipoles and hence the electric field (Figure S2b,c). This yields a disturbance in the electronic oscillations which can result in a change in the optical response and thus lead to the excitation of more than one plasmon mode (PM) [56,57,58]. The broadening of plasmon resonance indicates a broad distribution of particle sizes in the MLs. The reflection amplitude increases with the metal filling factor, which shows that the NP density is directly proportional to the absorption intensity [59]. Sequels to this plasmon are shifted to lower wavelength (blue shift) from sample Au/PPFC_1 to Au/PPFC_3. There are a number of reasons that can lead to the observed blue plasmon shift. The optical properties of nanocomposites are highly sensitive to the morphology and the surrounding dielectric medium. The decrease in particle size or increase in interparticle distance can result in a blue shift of the plasmon resonance [3,60,61]. In the present case, the Au NP size and interparticle distance in the three pristine samples have been found to be almost consistent (Table 3) with deposition and self-assembly into NP of 1 nm Au per layer thickness, which confirms that in pristine MLs, NPs are still surrounded by polymer, thus blocking the possible mobility of NPs. The shape is also found to be spherical (confirmed by GISAXS & GIWAXS), so we exclude these reasons for the plasmon shift in our system. In this case, among the possible causes for the plasmon shift is a possible variation in the refractive index of the polymer in the vicinity of the metal particles [7,49]. In the present study, we have varied the polymer thickness in the MLs; this may induce variation of the mass density of the polymer. Due to this, the polymer–filler bonding would vary; this structural change can alter the refractive index of the polymer [62,63]. Given this information, we think that in pristine Au/PPFC_2 and Au/PPFC_3 MLs, the splitting of the surface plasmon resonance is the effect of close coupling and varied polymer refractive index. The LSPR of gold NPs can be used to assess the binding energy. To quantify this, the indirect band gape energy of the three MLs was calculated using the Kubelka–Munck radiative transfer model [64,65,66].

(7) F(hν)=(F (Ref)×E)12,  F(Ref)=(1−Ref)22Ref

where $R_{ef}$ is the reflectance. The model allows the calculation of the reflectance from a layer that both scatters and absorbs light. The linear fit through the LSPR yields the indirect band gap energy of the system. Figure 6b depicts the plot of (F (Ref)×E)12 vs. energy for three MLs at room temperature and indicates a linear fit. The resulting extrapolated values are given in Table 4. From Table 4, one can see that the indirect band gap exhibits a systematic increase from sample Au/PPFC_1 to Au/PPFC_3. According to the Penn model [67], the dielectric constant of semiconductor materials [68,69] and other materials [70] varies inversely with the band gap energy. Thus, as one goes from Au/PPFC_1 to Au/PPFC_3, the dielectric constant is expected to decrease, which should result in blue-shift of the SPR, as observed in the present case, corroborating our hypothesis. These findings elucidate the nature of plasmon modes in this ML system, which involves strong light–matter coupling, and sets the level for the controlled bond formation by light excitation.

### 3.6. Temperature-Dependent Optical Response

The sensitivity of the nanocomposite film to the host medium was tested by monitoring the change in the Au-LSPR position as a function of temperature. The MLs were ex-situ annealed at 373 K, 473 K, and 573 K with the same annealing protocol used for GISAXS and GIWAXS measurements and their UV-Vis spectra were recorded. From Figure S7, one can see that in all the three cases the LSPR peak exhibits a blue shift with increasing annealing temperature. Furthermore, the magnitude of the shift decreases as one goes from sample Au/PPFC_1 to Au/PPFC_3 (Figure S8). Even a slight change from a nonspherical to a spherical shape can lead to a blue shift of the plasmon resonance [71,72]. The decrease in the magnitude of the blue shift in the three MLs is an indication of the reorganization of the NPs. The band width of the LSPR band of the annealed sample indicates only a small variation in the size distribution. In general, by annealing the samples well above the T_g_ of the polymer, the response to the dielectric environment significantly was enhanced. The increased sensitivity of the annealed sample is attributed to the increased mobility of both polymer chains and Au NPs in the close molten state of the polymer. The embedding of the Au NPs changed from sparse to a denser form due to a successive decrease in polymer thickness in the three MLs (Figure S2). In addition to this, the NP size increases with thermal annealing. Larger particles move slowly and this is why the magnitude of the shift is decreased from Au/PPFC_1 to Au/PPFC_3 (Figure S8). The results obtained are summarized in Table 4. Thus, the optical response of NPs during thermal treatment can possibly be explained in terms of the joint effect of effective refractive index variation and the depolarization factor as the particles come closer and there is a possibility of a slight change in shape (depolarization factor also contributes due to the small neck (rod-like shape) between two spherical particles) [73].

The result shows that, by using adequate postdeposition annealing, tailoring of the optical properties of such a system is possible and could be preferential for plasmatic-driven applications. From Table 4, one can see an increase in the band gap energy with increasing temperature. This shows a higher mismatch of the crystal momentum in the valence and conduction bands [74]. Qualitatively, according to the Penn model, this demonstrates the decrement of the dielectric constant (refractive index). An increase of the dielectric constant leads to a shift of the absorption maximum towards longer wavelengths [75,76]. However, the present results are in conformity with a decreasing dielectric constant, as the dielectric constant is supposed to decrease from Au/PPFC_1 to Au/PPFC_3, which leads to a blue shift of the plasmon resonance.

## 4. Conclusions

A simple, practical approach has been developed to make nanoengineered ML structures that can control the optical response of the NPs. A well-defined in-plane interparticle correlation is observed in all the three pristine MLs with an average distance of 4.30 ± 0.05 nm (Table 1), in conformity with simulations. Furthermore, a strong interlayer-particle correlation is observed in the vertical direction, in agreement with the bilayer period variation in the three MLs (Figure S2; Table 2). The reordering of NPs occurs as an effect of thermal annealing. The dependence of the NPs’ size on the square root of the annealing time suggests diffusion-controlled growth of NPs. These findings indicate that the mobility of metal NPs can be affected by the volume fraction of metal in a polymer and the following annealing. Enhanced diffusion and intermixing is the main reason for the structural changes. It is worth noting that after annealing at 573 K, the interparticle correlation became more isotropic in both in-plane/horizontal and vertical directions. At the same time, NP size increases with increasing temperature and their shape becomes more spherical due to a reduction in the surface energy. The stability limit can be shifted to higher temperature by varying the intermediate polymer layer thickness, which can be useful in device applications. UV-Vis analysis shows that the LSPR frequency exhibits systematic variation with the volume fraction and thermal annealing. The variation in the dielectric constant of the material is found to be a possible factor affecting the LSPR frequency. The structural properties of the MLs are found to be in good agreement with their optical responses. Thus, the optical properties are tunable with appropriate choice of thermal treatment and volume fraction of metal in a favorable polymer matrix. This work opens the way to tune ML optical properties via controlling the growth of metal NPs.

## Figures and Tables

**Table 1:** Au and polymer layer thicknesses, minimum crystalline size (111) (*D_np_*), lattice constant (a), in-plane interparticle distance ($\xi_{H}$) of all three multilayers at room temperature. Errors indicated only in the first line hold for all values.

Sample	*δ*(Au) (nm)	*δ*(PPFC) (nm)	*D_np_* (nm)	a (nm)	$\xi_{H}\;(\mathrm{nm})$
Au/PPFC_1	1 ± 0.1	19 ± 0.8	2.43 ± 0.004	0.403 ± 0.001	4.30 ± 0.05
Au/PPFC_2	1	9	2.46	0.404	4.34
Au/PPFC_3	1	5.6	2.45	0.406	4.32

**Table 2:** Interlayer-particle distance ($\xi_{V}$) calculated from the first order Bragg peak of the multilayers at various temperatures. The errors only given in one row hold for all three MLs with varying temperature. n.q.: not quantifiable.

Multilayer	$\xi_{V}\;(\mathrm{nm})\;273\;K$	$\xi_{V}\;(\mathrm{nm})\;373\;K$	$\xi_{V}\;(\mathrm{nm})\;473\;K$	$\xi_{V}\;(\mathrm{nm})\;573\;K$
Au/PPFC_1	10.45 ± 0.03	9.79 ± 0.04	9.18 ± 0.07	5.13 ± 0.1
Au/PPFC_2	6.86 ± 0.03	6.61 ± 0.04	4.65±0.07	n.q.
Au/PPFC_3	3.82 ± 0.03	7.02 ± 0.04	n.q.	n.q.

**Table 3:** NPs radius (*R*) and interparticle distance ($\xi_{H})$ variation in three MLs with varying temperature. The values for the errors given in row 1 hold for all values in the columns.

Temperature	MLs	$\xi_{H}\;(\mathrm{nm})$	*R* (nm)
RT	Au/PPFC_1	4.30 ± 0.05	1.56 ± 0.003
	Au/PPFC_2	4.34	1.57
	Au/PPFC_3	4.32	1.57
373 K	Au/PPFC_1	5.20	1.67
	Au/PPFC_2	5.81	1.91
	Au/PPFC_3	5.92	1.93
473 K	Au/PPFC_1	6.92	2.02
	Au/PPFC_2	6.03	1.96
	Au/PPFC_3	6.31	2.01
573 K	Au/PPFC_1	6.48	1.94
	Au/PPFC_2	5.89	1.93
	Au/PPFC_3	6.35	2.03

**Table 4:** Evaluated indirect band gap energy (in eV) for three multilayers at different temperatures.

Sample	RT	373 K	473 K	573 K
Au/PPFC_1	2.51 ± 0.04	2.61 ± 0.03	2.67 ± 0.05	3.18 ± 0.03
Au/PPFC_2	2.58 ± 0.04	2.82 ± 0.03	2.94 ± 0.05	3.70 ± 0.03
Au/PPFC_3	2.66 ± 0.04	2.85 ± 0.03	2.93 ± 0.05	2.95 ± 0.03

## Supplementary Materials

The following are available online at https://www.mdpi.com/2079-4991/9/9/1249/s1, Figure S1: Schematic diagram of alternate sputter deposition, Table S1: Filling factor, density of composite and volume fraction from the 3 MLs, Figure S2: Schematic structure presentations of the three MLs, the layer waviness is because of Au inclusion in PPFC matrix (a–c), 1D GIWAXS, GISAXS–yoneda and off detector cut, Figure S3: The sketch of GISAXS/GIWAXS experiment at Elettra BL 5.2 illustrates the angles and the distance used, Figure S4: Bilayer Simulation in ML Au/PPFC_1, Figure S5: Minimum crystalline size variation with time and temperature in MLs (a) Au/PPFC_1, (b) Au/PPFC_2 MLs and their corresponding Arrhenius-plot, Figure S6: Particle size variation with time and temperature in MLs a)Au/PPFC_1, b) Au/PPFC_2 MLs and their corresponding Arrhenius-plot, Figure S7: UV-Vis reflectance spectra of MLs Au/PPFC_1 (a), Au/PPFC_2 (b) and Au/PPFC_3 (c) at different temperatures, Figure S8: F(Ref)×(h*ν)^1/2^ as a function of photon energy for a)Au/PPFC_1, b)Au/PPFC_2 and c)Au/PPFC_3 at varying temperature.
