# Supplementary material for: Structure–Function Correlations in Sputter Deposited Gold/Fluorocarbon Multilayers for Tuning Optical Response
Source: Nanomaterials (Basel). 2019 Sep 3;9(9):1249. doi: 10.3390/nano9091249 (PMC6780487; doi:10.3390/nano9091249)
Supplement: Supplementary file 1 [file nanomaterials-09-01249-s001.pdf]

Structure-function correlations in sputter deposited gold/fluorocarbon multilayers for tuning optical response.

Supplementary Materials

General:

Different Au/ polymer multilayer structure have been prepared by varying the Au volume fraction. The volume fraction of Au in PPFC has been calculated for a fixed filling factor (taken from literature[1]) using the following equations. The calculated values for three MLs have been listed in Table (S1).

Filling factor (F):

$$ff = \frac{\rho - \rho_p}{\rho_m - \rho_p} \quad (1)$$

here, ρ is the density of nanocomposite and ρ_p, ρ_m are the densities of the polymer and metal, respectively.

Composite density: ϱ = m/v

$$\rho = \frac{m_m + m_p}{V_m + V_p} \quad (2)$$

where V_p and m_p is volume and mass of the polymer and V_m and m_m are the volume and mass of the metal.

Volume fraction:

$$\frac{V_m}{V_p} = \frac{\rho - \rho_p}{\rho_m - \rho} \quad (3)$$

Table S1: Filling factor, density of composite and volume fraction from the 3 MLs.

Multilayer	ff	ϱ g/cm^3	$\varphi = V_m/V_p$
Au/PPFC_1	0.12	3.19±0.1	0.13±0.02
Au/PPFC_2	0.27	4.44	0.37
Au/PPFC_3	0.38	5.35	0.61

Schematic sketch of sample preparation through sputtering technique:

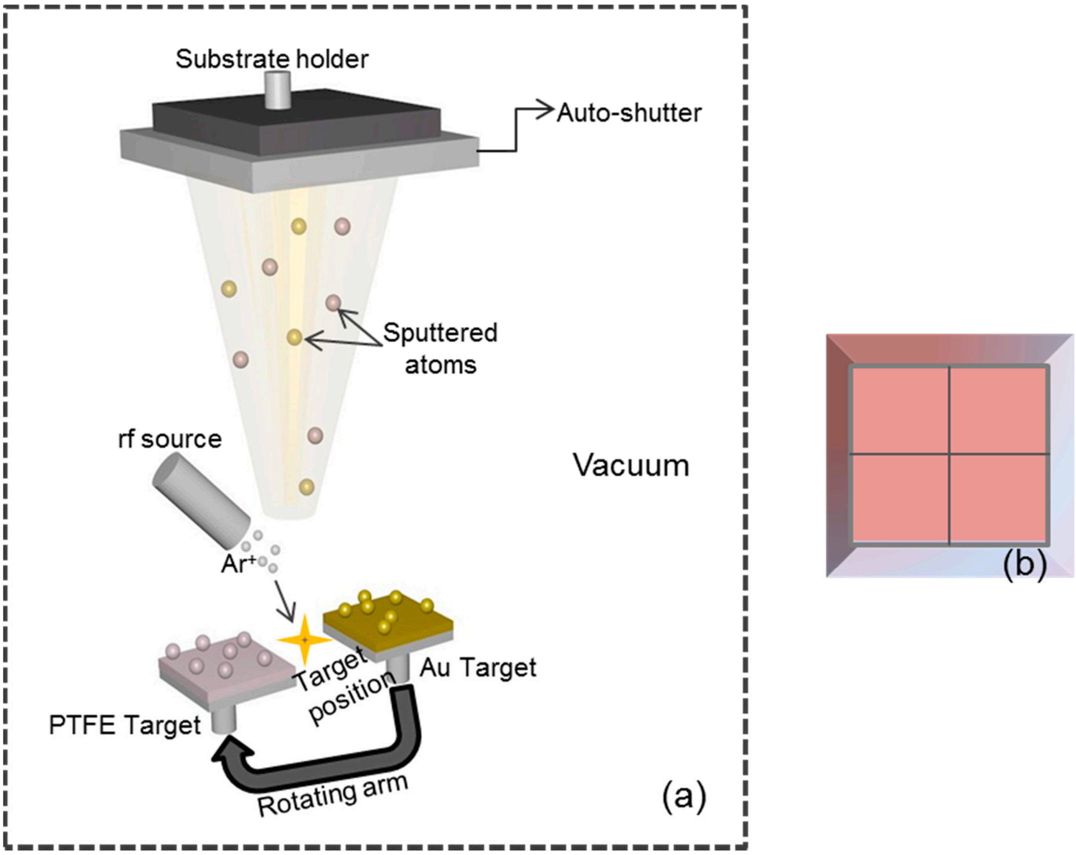

Figure S1. a) Schematic diagram of alternate sputter deposition of Au and PPFC. A rotating arm was used to switch the target. Target position was fixed in forward direction of ion beam. An auto shutter was used to cover the substrate surface during change of targets and b) Prepared sample of size of 50 × 50 mm^2, the most uniform region in the middle 40 × 40 mm^2 has been cut and used for various measurement.

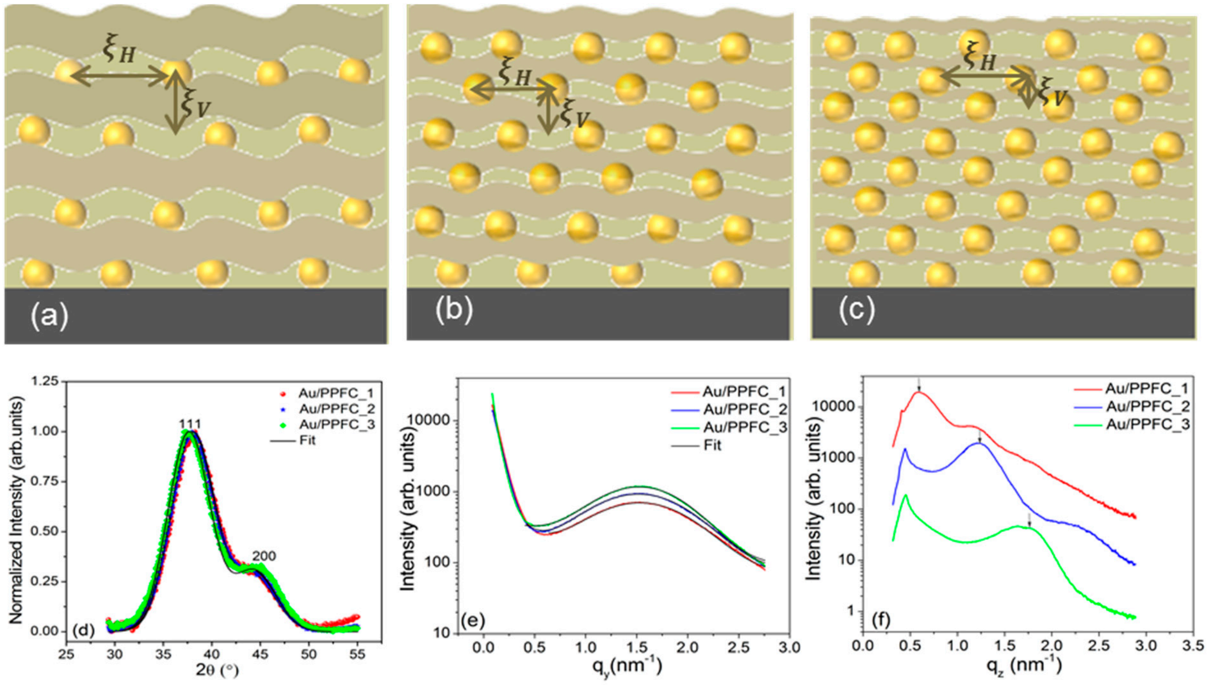

Figure S2. Schematic structure presentations of the three MLs a) Si/[Au(1nm)/PPFC (19nm)], b) Si/[Au(1nm)/PPFC (9nm)] and c) Si/[Au(1nm)/PPFC (5.6nm)], the layer waviness is because of Au inclusion in PPFC matrix , d) Normalized 1D GIWAXS pattern of three pristine MLs and black lines represent their corresponding fit result (black line), e) GISAXS 1D pattern of pristine MLs as a function of $q_{y,}$ and f) The off-detector cuts of the three pristine MLs shifted vertically for better visibility of the individual features.

Sketch of detector positioned used for the experiment:

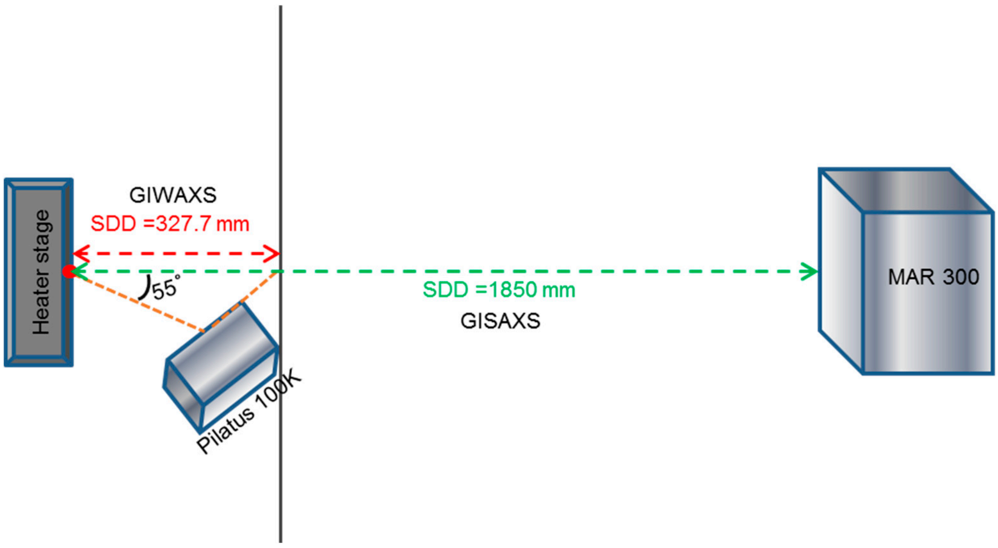

Figure S3. The sketch above illustrates the angles and the distance used in GISAXS/GIWAXS experiment at Elettra BL 5.2.

IsGISAXS simulation:

A simulation of the GISAXS patterns of the sample Au/PPFC_1 was performed using the software IsGISAXS[2]. Its result was used as reference in further calculations since the morphology and structure in each individual layer of the multilayer (ML) system is identical. Modeling was limited to lateral correlations and the system Si/[Au(1 nm)/PPFC(19 nm)], .i.e the bottommost layer comparable to the Au/PPFC_1 using Distorted Wave Born approximation DWBA. We used a 1D paracrystal interference function with a distance ξ_H = 4.3± 0.05 nm and a disorder factor ω/ξ_H = 0.33. We used the Local mono-disperse approximation (LMA) and lognormal distribution in radius with σ=1.08 and R_{min} = 1.2 nm and R_{max}= 2 nm. Incident angle and wavelength were set according to the experimental values. δ and β of the complex index of refraction $\eta = \delta + i\beta$ of the different materials involved are given below.

Material	Layer thickness (nm)	δ	β	RMS roughness
Si_substrate	5	7.6462×10^{-6}	1.7567×10^{-7}	2
Au	1	4.7559×10^{-5}	4.9278×10^{-6}	
PPFC	19	6.8741×10^{-6}	3.45972×10^{-8}	

This model assumption allowed for correlating the side maximum in q_y-space with a real-space distance ξ_H and subsequently to apply the spherical geometrical model, based on a modification using spherical particles in geometrical model by Schwartzkopf *et al.*[3]

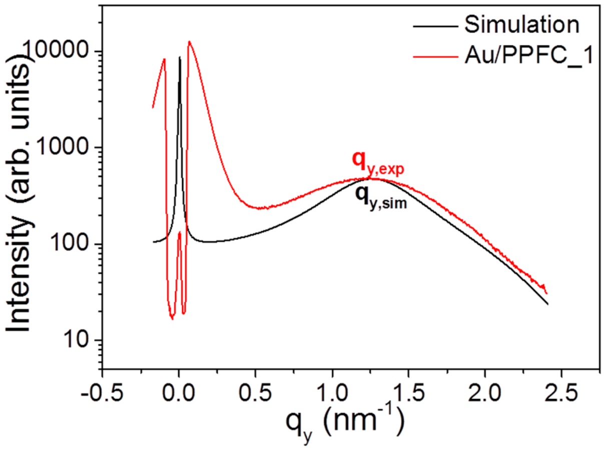

Figure S4. Comparison of simulated and experimental GISAXS pattern for Si[Au(1nm)/PPFC(19nm)]. The simulation data were scaled to the intensity of the side maximum in the experimental data.

In figure S6, the side peak position and fall-off of the curve at higher q_y-values due to the shape of the NP are well produced. Discrepancies at small q_y-value stem from the fact that only one single layer

system could be simulated, and experimental resolution was not included. Nonetheless, this simulation allows for quantitatively extracting the maximum values and to verify the use of the modified geometrical model by Schwartzkopf [3]. The position of the side maximum $q_{y,exp}$ in the experimental data was extracted by fitting a Gaussian function yields the real-space length $\xi_{H,exp} = \frac{2\pi}{q_{y,exp}}$ (~4.7 nm in this case). From the simulation, $\xi_{H,sim} = \frac{2\pi}{q_{y,sim}}$ (in this case ~4.3 nm) was calculated, thus allowing to refine the real-space length scale taking into account refraction/reflection effects This allows for directly relating the position of the side maximum in q_y-space to the real-space interparticle distance obtained by simulation and shows that the real-space length scale is reduced by the refinement factor 4.3/4.7. The position of the side maximum was extracted for all other samples at various temperatures using accordingly DPDAK [4] (Gaussian fit) and the real space interparticle distance was refined based on results from simulation reference and the refinement factor. This procedure allows for the rapid analysis of GISAXS data taking the refraction/reflection effects into the account.

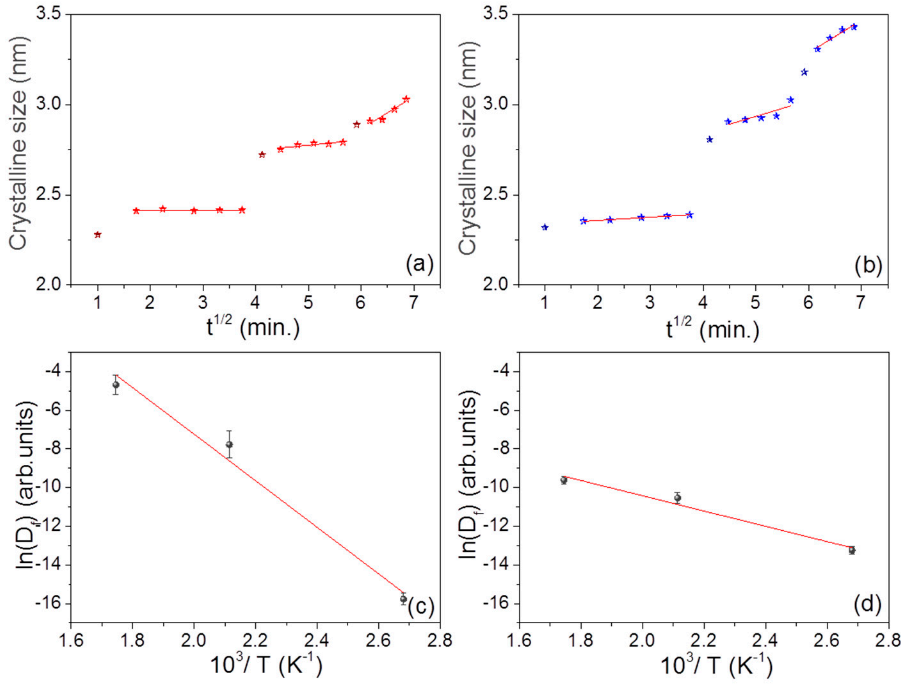

Figure S5. Minimum crystalline size variation with time and temperature in MLs a) Au/PPFC_1, b) Au/PPFC_2 MLs and their corresponding Arrhenius-plot c) Au/PPFC_1, d) Au/PPFC_2.

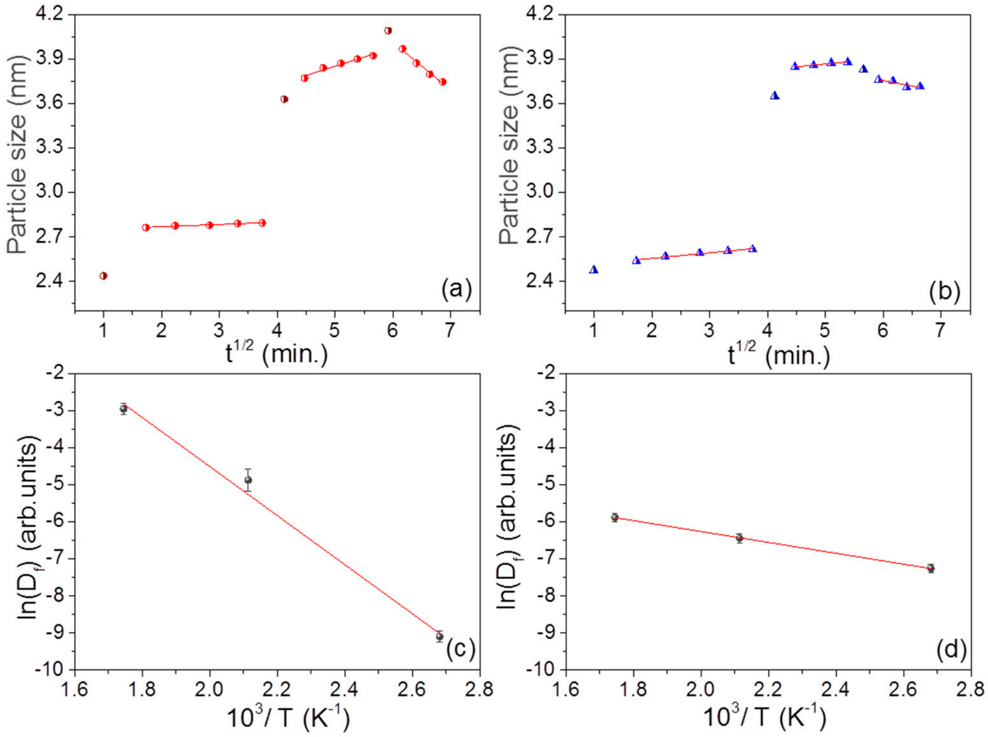

Figure S6. Particle size variation with time and temperature in / MLs a) Au/PPFC_1, b) Au/PPFC_2 and their corresponding Arrhenius-plot c) Au/PPFC_1. and d) Au/PPFC_2.

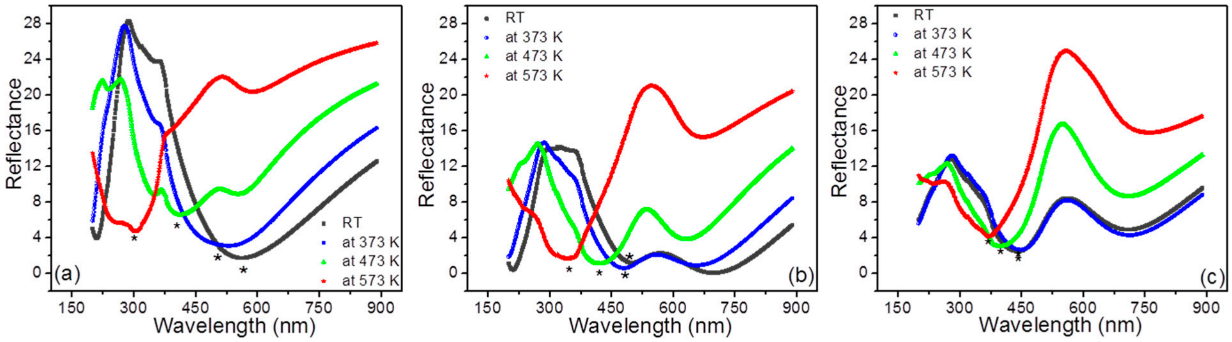

Figure S7. UV-Vis reflectance spectra of Au/PPFC_1, (a) Au/PPFC_2 (b) and Au/PPFC_3 (c) at different temperatures. LSPR positions in three MLs are indicated by symbol (*).

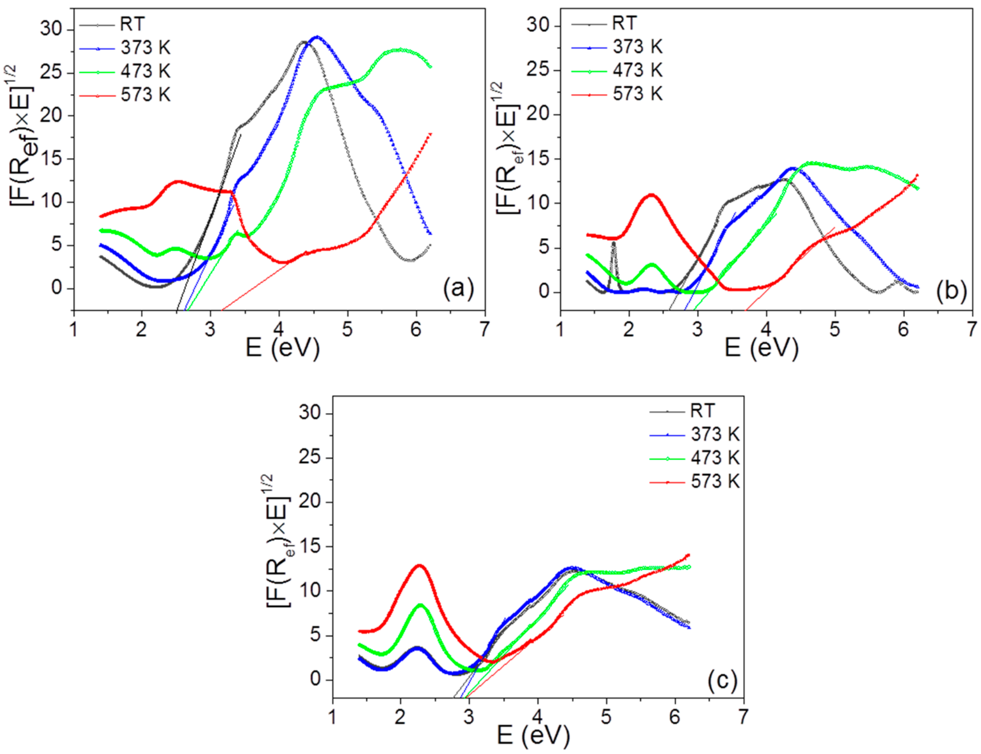

Figure S8. $F(R_{ef})\times(h^{*}\nu)^{1/2}$ as a function of photon energy for a) Au/PPFC_1, b) Au/PPFC_2 and c) Au/PPFC_3 at varying temperature.

References

1. Takele, H.; Jebril, S.; Strunskus, T.; Zaporojchenko, V.; Adelung, R.; Faupel, F. Tuning of electrical and structural properties of metal-polymer nanocomposite films prepared by co-evaporation technique. *Applied Physics A: Materials Science and Processing* **2008**, *92*, 345–350.

2. Lazzari, R. IsGISAXS: A program for grazing-incidence small-angle X-ray scattering analysis of supported islands. *Journal of Applied Crystallography* **2002**, *35*, 406–421.

3. Schwartzkopf, M.; Buffet, A.; Körstgens, V.; Metwalli, E.; Schlage, K.; Benecke, G.; Perlich, J.; Rawolle, M.; Rothkirch, A.; Heidmann, B.; et al. From atoms to layers: in situ gold cluster growth kinetics during sputter deposition. *Nanoscale* **2013**, *5*, 5053.

4. Benecke, G.; Wagermaier, W.; Li, C.; Schwartzkopf, M.; Flucke, G.; Hoerth, R.; Zizak, I.; Burghammer, M.; Metwalli, E.; Müller-Buschbaum, P.; et al. A customizable software for fast reduction and analysis of large X-ray scattering data sets: Applications of the new DPDAK package to small-angle X-ray scattering and grazing-incidence small-angle X-ray scattering. *Journal of Applied Crystallography* **2014**, *47*, 1797–1803.
